# Prescribing Patterns of Pain Medications in Unspecific Low Back Pain in Primary Care: A Retrospective Analysis

**DOI:** 10.3390/jcm10071366

**Published:** 2021-03-26

**Authors:** Stefania Di Gangi, Giuseppe Pichierri, Stefan Zechmann, Thomas Rosemann, Andreas Plate

**Affiliations:** Institute of Primary Care, University and University Hospital Zurich, 8091 Zürich, Switzerland; giuseppe.pichierri@usz.ch (G.P.); stefan.zechmann@usb.ch (S.Z.); thomas.rosemann@usz.ch (T.R.); andreas.plate@usz.ch (A.P.)

**Keywords:** low back pain, pain medication, NSAID, opioid, primary care

## Abstract

Acute low back pain (LBP) is one of the most prevalent diseases worldwide. Since there is evidence of excessive prescriptions of analgesics, i.e., opioids, the aim of this study was to describe the use of pain medications in patients with LBP in the Swiss primary care setting. A retrospective, observational study was performed using medical prescriptions of 180 general practitioners (GP) during years 2009–2020. Patterns of pain medications (nonsteroidal anti-inflammatory drugs (NSAIDs), paracetamol, and opioids) as well as co-medications were analyzed in patients with a LBP diagnosis. Univariable and multivariable regression analyses assessed GP and patient characteristics associated with the prescription of pain medication. Patients included were 10,331 (mean age 51.7 years, 51.2% female); 6449 (62.4%) received at least one pain medication and of these 86% receive NSAIDs and 22% opioids. GP characteristics (i.e., self-employment status) and patient characteristics (male gender and number of consultations) were associated with significantly higher odds of receiving any pain medication in multivariable analysis. 3719 patients (36%) received co-medications. Proton-pump-inhibitors and muscle relaxants were the most commonly used co-medications. In conclusion, two-thirds of LBP patients were treated with pain medications. Prescribing patterns were conservative, with little use of strong opioids and co-medications.

## 1. Introduction

Low back pain (LBP) is one of the most prevalent diseases worldwide [1]. The lifetime prevalence of LBP in developed countries is up to 80% [2,3,4,5] and a systematic review calculated the incidence ranged between 0.024 and 7.0% [6]. In Switzerland 43% of the population report an episode of LBP within the last four weeks [4] and back pain ranked second in patient reported reasons for physician consultations [7].

Despite the often self-limiting and benign character of LBP [4,8], the individual burden in some patients can be high and many patients still receive supportive therapies [9]. Key driver for a treatment is the intention to reduce the individual burden and to prevent chronicity. The patterns of pharmacological treatment are of interest and numerous national and international treatment recommendations or guidelines have been developed to provide high quality of care in pharmacological patient treatment. However, there is a considerable degree of disagreement between the different recommendations [10] and guideline adherence varies [3,9,11]. Therefore, prescribing patterns vary between the different countries and different health care settings [9,12,13,14]. Of particular interest is the use of opioids in non-cancer patients, as their use is associated with increased rates of side effects like dependence or substance abuse, as seen in the “opioid-crisis”, in North America [15,16].

The focus of this study was the prescription of pain medications for the treatment of LBP in the Swiss primary care sector. In particular, we aimed to determine the number of patients with LBP treated with at least one pain medication and to examine the patterns of prescriptions. We sought to determine prescriber and patient characteristics associated with higher odds of receiving pain medication. Finally, we aimed to determine frequencies and patterns of prescribed co-medications in the treatment of acute LBP.

## 2. Materials and Methods

### 2.1. Study Design and Database

This observational study was retrospective. Data were extracted from the FIRE (Family medicine ICPC Research using Electronic medical records) database. The database, since 2009, collects routine medical data from around 500 General practitioners (GPs), around 10% of all GPs in the German-speaking area of Switzerland. Currently it holds records of >780,000 patients and >9.4 million consultations.

### 2.2. Inclusion Criteria

**Figure 1:**
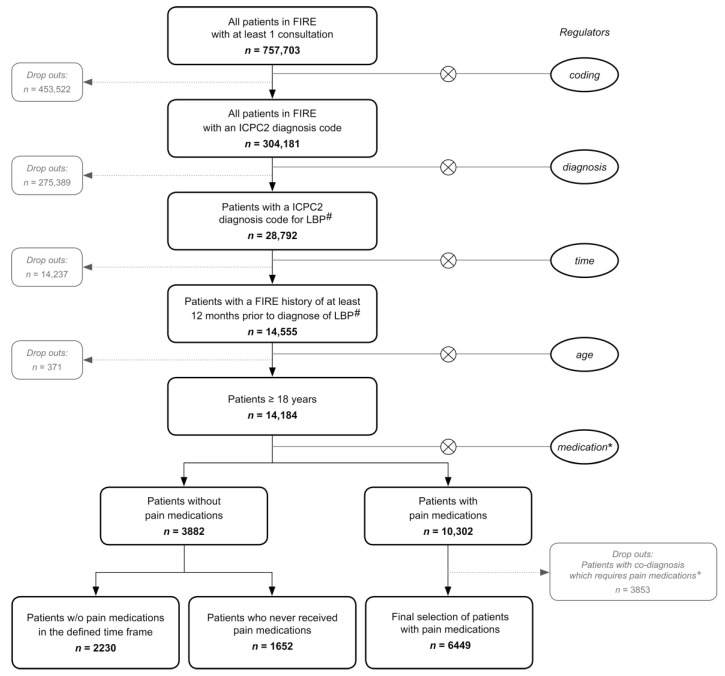
Flowchart. FIRE: Family medicine ICPC Research using Electronic medical records; *n*: number of patients; w/o: without; #: ICPC2 LBP diagnosis codes: L03, L84, L86; *: Pain medication of interest: non-steroidal anti-inflammatory drugs, paracetamol and opioids; +: Full list of all excluded ICPC2 diagnosis codes in Supplementary Material.

The study period was 2009–2020. According to the current International Classification of Primary Care 2 (ICPC-2) diagnosis code system LBP was defined as: (1) L86-Back syndrome with radiating pain; (2) L84-Back syndrome without radiating pain; (3) L03-Low back symptom/complaint. All patients ≥ 18 years with a LBP diagnosis and at least one consultation within the twelve months (baseline period) prior to the LBP diagnosis (Figure 1) were included. Then they were followed-up for six months after diagnosis. Only the first episode of LBP and all consultations from the day of diagnosis were considered. For the analysis of pain medications and co-medications, patients with specific ICPC-2 codes, which would independently qualify for a pain medication (for example cancer or trauma patients) were excluded (full list in Supplementary Material).

### 2.3. Study Variables

Pain medications of interest were nonsteroidal anti-inflammatory drugs (NSAIDs), paracetamol, and opioids. Prescription patterns were described in terms of percentages of patients with one or more prescriptions. Time of prescriptions was defined as a continuous variable (in month unit) or categorized in two ways, as necessary: (1) before diagnosis, at diagnosis, within week 1 after diagnosis, within week 2–4 after diagnosis, later than four weeks after diagnosis; (2) before diagnosis, at diagnosis, 0–2 months after the diagnosis, 2–6 months after the diagnosis.

To determine patient and prescriber characteristics associated with prescriptions, we used the following characteristics: patient related (age at diagnosis ≤ or > 50 years, sex, number of consultations after diagnosis, LBP syndrome groups, or diagnosis code) and GP related (sex, age: continuous or binary with cut-off 55 years, years of experience in practice, type of practice, type and level of employment, self-dispensing of drugs). Type of practice was defined as: single, double or group practice. Type of employment categories were: self-employed or employee. Level of employment was defined as: <50%, between 50 and 79%, between 80 and 99% and 100%. Drug dispensing was considered as a possible factor that might influence prescriptions at GP level. In fact in some regions of Switzerland, GPs are allowed to dispense drugs, but not in others, therefore the variable drug dispensing was defined as yes or no.

Co-medications of interest were: proton-pump-inhibitors (PPI), antidepressants, sleeping pills, and muscle relaxants. All medications were analyzed using the Anatomical Therapeutic Chemical Classification System (ATC) codes. All ATC codes are provided in Supplementary Material.

### 2.4. Statistical Analysis

Summary statistics were reported as means (standard deviation, SD) and range (min–max), median (interquartile range, IQR) or number (percentage, %) as appropriate. In descriptive tables prescription patterns were compared between the LBP syndrome groups using one-way ANOVA test and chi-square test, with/without simulated *p*-values, as appropriate. Patients with code combinations (for example patients with simultaneous codes for “Low back symptom/complaint” and “Back syndrome without radiating pain”) were excluded for group comparisons. Analogously, co-medications use was also compared between these groups.

Prescription rates of pain medications were represented graphically with the density (frequency distribution of prescribed pain medications in density scale) and cumulative (cumulative frequency of prescribed pain medications) functions. Counts of prescriptions per patient, overall and stratified by LBP groups, were modelled through Poisson regression models corrected for repeated measurements within patients. Results were presented as risk ratio (RR) (95% confidence interval (CI)).

Univariable and multivariable mixed-effects logistic regression analyses were performed to assess the association of GP and patient determinants with any pain medication prescription and, as separate analyses, any NSAIDs, opioids and Paracetamol prescriptions. A stratified analysis by LBP groups was also performed. Mixed models were used to account for the correlation of patients within GP and were performed for any and specific prescriptions. In univariable analysis, every effect was considered separately in a single model. In multivariable analysis, relevant characteristics were considered together. Multivariable models were developed starting from variables with *p* ≤ 0.2 in univariable analysis and then implementing a stepwise backward elimination to include all relevant factors which better fit the models. Missing observations were removed from the analysis. Results of regression analyses were presented as odds ratio (OR) (95% CI) with indication of the number of non-missing observations, in terms of patients and GP. Intra-class correlation (ICC) was also reported in multivariable analysis. For all tests, *p* ≤ 0.05 was considered statistically significant. All analyses were carried out using statistical package R version 3.6.1. (https://www.R-project.org, accessed on 25 March 2021).

### 2.5. Ethics

Research using the FIRE database did not need ethical approval, as the project did not fall under the scope of the Federal Act on Research involving Human Beings (Human Research Act) (BASEC-Nr: Req-2017-0079).

## 3. Results

### 3.1. Basic Characteristics

We included 10,331 patients (Figure 1). 5289 (51.2%) patients were female. The mean age was 51.7 years (range: 18–100 years). A number of 8222 patients (80%) had a diagnosis code of non-radiating back pain (L03 or L84 plus 94 patients with a combination of L03 and L84). On average, each patient had 2.1 consultations (median: one consultation (IQR: 1.00, 2.00)). Between groups, a significant difference was found in the distribution of consultations, age, and sex. In particular, patients with radiating pain were significantly older and had, on average, more consultations compared to patients in the diagnostic groups with non-radiating pain. Furthermore, comparing the patients with a back syndrome with radiating and without radiating pain, we found a higher proportion of female patients in the group with radiating pain. (Table 1).

### 3.2. Prescribing Patterns

Patterns of prescribed pain medications were presented in Table 2: 62.4% (6449 patients) received at least one pain medication and 25.4% (2623 patients) received two or more pain medications. Of the patients with at least one pain medication, the proportion of patients receiving NSAIDs, paracetamol, and opioids was 86% (5545 patients), 39.4% (2544), and 22% (1417), respectively. The most commonly used NSAID was diclofenac (2502 prescriptions), and the most commonly prescribed opioid was tramadol (1180 prescriptions). A complete list of prescribed pain medications was provided in Table S1, Supplementary Material. Patients with an opioid therapy had in most cases a concurrent therapy with a NSAID and/or paracetamol (87.4%) and seldom opioids alone (12.6%). There were significantly fewer pain medication prescriptions in the group “Low back symptom/complaint” compared to the group “Back syndrome without radiating pain “(*p* < 0.001) (Table 2). Among patients with medication, 1.84 pain medications were prescribed on average. We found no relevant difference in the number of pain medications prescribed in dependence of the time of prescription (Table S2, Supplementary Material).

### 3.3. Time Patterns of Prescriptions

**Figure 2:**
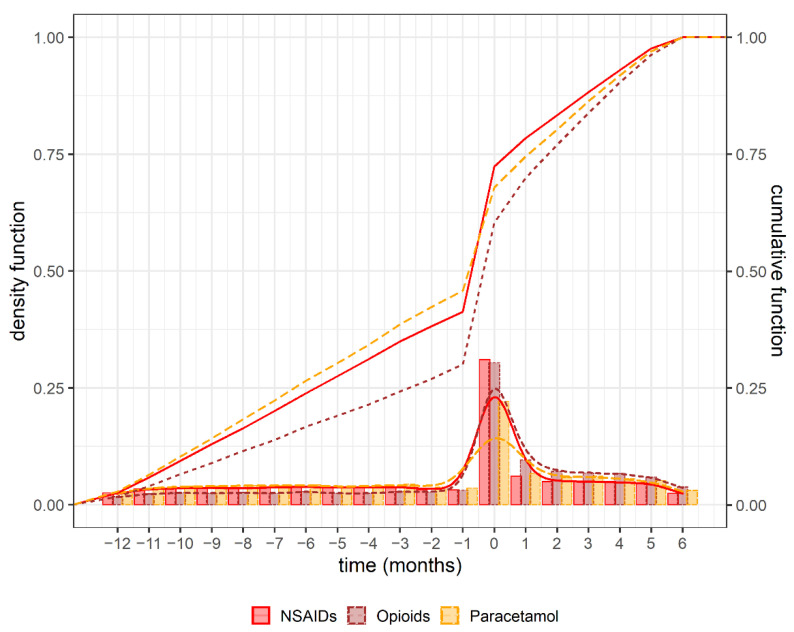
Time pattern of prescriptions: This figure shows the time pattern of pain medication prescriptions in 6449 patients during baseline and follow up period, in months. Primary y-axis: histogram in scale density (density function, lower curves) and its smoothed curve for each pain medication class. It describes the proportion of prescriptions occurring in that time period. Secondary y-axis: cumulative proportion (cumulative function, upper curves) of all pain medication prescriptions over time. It describes the total prescription rate up to the time period. NSAIDs: non-steroidal anti-inflammatory drugs.

During the baseline period, we observed a consistent rate of prescriptions, and nearly 50% of NSAIDs were prescribed before diagnosis (Figure 2, cumulative function). After the peak at time of diagnosis, pain medication prescriptions, still elevated in the follow up period, declined back to the level of prescribing during the baseline period (Figure 2—density function and Table S3, Supplementary Material). In patients with only pain medications at or after the diagnosis, the average of the time from diagnosis to the first NSAID, paracetamol, or opioid prescription was 9, 13 and 8 days, respectively (Table 2, part C). However, in this group the majority of pain medications were prescribed at the day of diagnosis (median 0.0, (IQR: 0.00, 0.00) for all groups). Prescribing patterns in patients with multiple prescriptions of one or different classes is shown in Table S4, Supplementary Material. In patients with a NSAID/opioid co-medication, the first opioid prescription in this group was on average 9 days after the first NSAID.

The models of the number of prescriptions per patient, overall and by LBP diagnosis groups were reported in Table S5, Supplementary Material. After correcting for the type of medication, patients with Low back symptom/complaint and prescriptions after two months from the diagnosis had a higher risk of getting more prescriptions, compared to the ones who got prescriptions at diagnosis (RR = 1.06, 95% CI: 1.01–1.11, *p* = 0.017). Moreover, after correcting for time of prescriptions, in all LBP subgroups Opioids and Paracetamol have a lower incidence rate compared to NSAIDS.

### 3.4. Regression Analysis: GP and Patient Characteristics Association

Associations between patient and GP characteristics on pain medication prescription (any pain medication and by pain medication subgroup) are shown in Table 3. Additional regression analysis, univariable and multivariable, stratified by LBP diagnosis group was reported in Table S6, Supplementary Material. We identified both GP (self-employment and non-self-dispensing status) and patient characteristics (male gender and number of consultations) which were associated with significantly higher odds of receiving any pain medication in multivariable analysis. In subgroup analysis for each pain medication class we found that, after correcting for other confounding factors, patient age > 50 years, at the time of diagnosis, was significantly associated with increased odds of prescribing NSAIDs (OR: 1.24, 95%CI: 1.13–1.36, *p* < 0.001), but decreased odds of prescribing opioids (OR: 0.66, 95%CI: 0.58–0.76, *p* < 0.001) or paracetamol (OR: 0.84, 95%CI: 0.75–0.93, *p* = 0.001).

### 3.5. Co-Medications

3719 patients (36% of 10,331 patients) received one or more co-medications of interest and had no other ICPC-2 diagnosis codes, which would qualify for one of the co-medications (Table S7, Supplementary Material). Patients with a radiating pain had more co-medications compared to the other groups (45.5% vs. 35.4%, and 35.7%, respectively *p* < 0.001). Of all patients, the co-medications prescribed were: 12.7% (muscle relaxants), 7% (antidepressants), 6.4% (sleeping pills), and 22.4% (PPI), respectively, with pantoprazole and tizanidine the most commonly used drugs. Only one-third (31.7%) of patients with a NSAID therapy had a co-medication with a PPI.

## 4. Discussion

In this study, we determined frequencies and patterns of pain medication prescriptions in patients with LBP in Swiss primary care. Only two-thirds of all patients with an ICPC-2 diagnosis code of LBP were managed with any kind of pain medication therapy and if a pain medication was prescribed, in 86% of these cases at least a NSAID was prescribed. However, opioids were prescribed in one-fifth of all patients.

### 4.1. Pattern of Prescriptions

Nearly two-thirds of all patients in our cohort received at least one pain medication, with diclofenac, ibuprofen, and paracetamol being the most commonly used drugs. This proportion is similar to pain medication prescription rates from studies conducted in the US or The Netherlands [11,17]. However, there are considerable differences in the type of pain medication used. In our analysis, only 22% received opioids, with tramadol the most commonly used opioid. This is in contrast to the US, where recent studies, using health claims data or medical health records data, suggest that opioids are the most commonly used pain medications for LBP [11] or that nearly two-thirds of patients with a LBP diagnosis had taken opioids in the year before/after the diagnosis [18]. Opioid use is much more common in the US and Canada than in the European or Asian countries and per capita consumption has risen in the last years [19]. Most guidelines on LBP recommend the use of opioids only if non-opioids treatments have failed, as opioid use is associated with increased risk of dependency. A recent review found a pooled incidence of 4.7% for opioid dependency in patients treated with opioid for non-cancer pain [20]. In addition, opioid therapy in LBP patients is associated with increased odds of chronic work loss [21] and disability [22]. The proportion of patients receiving opioids in our cohort is still high, but two facts support a prudent use of opioids in the Swiss primary care setting. First, in our cohort, only 12.6% of patients with opioids lack a basic non-opioid pain therapy and second, tramadol, a weak opioid, was by far the most commonly prescribed opioid. This is in contrast to the data obtained in the US, where especially the use of strong narcotic opioids predominates the pain management [11]. However, we found that opioid treatment in our cohort was more common in patients ≤ 50 years of age. However, if opioids are prescribed in elderly patients, GPs should be aware of the higher risks of opioid associated side effects in this patient group [23] due to the higher prevalence of polypharmacy or comorbidities.

In real life, however, there is a difference between pain medication prescribed and pain medication taken by the patients. Back pain is one of the most common reasons for taking over the counter (OTC) pain medication [24]. Due to their free availability, ingestion mainly concerns the pain medications of the NSAID group and paracetamol. The pain medication taken in real life are therefore likely to be higher than those calculated in this study. In addition, there seems to be evidence, that not only quantity is affected by OTC medications, but also quality. For example in one study analyzing analgesic use in older adults with LBP, paracetamol appears to be obtained directly much more frequently than NSAIDs [25].

### 4.2. Time Pattern of the Prescriptions during Baseline and Follow-Up Period

Pain medications are recommended in acute LBP [26] and in accordance with the guidelines, we found a peak of pain medication prescriptions at time of diagnosis and the elevated level of pain medication consumption in the follow up period declined back to the level of prescribing during the baseline period. In addition, our observation, that NSAID prescriptions took place earlier in the course of LBP compared with opioids, are in line with many current recommendations [10,17,27,28,29,30] and confirm the overall restrictive use of opioids in Swiss Primary Care.

### 4.3. Association with Pain Medication Prescriptions

We identified both patient and GP characteristics associated with increased odds of pain medication prescriptions. To our knowledge, there are no studies analyzing specific patient or GP characteristics associated with prescribing patterns for acute LBP in the outpatient setting. However, pain in general is a strong predictor for the use of healthcare services [31]. For patients with chronic LBP in the primary care setting, it was reported that men, especially those with a high number of consultations, have not only a high number of prescribed medications but also higher opioid doses [18]. This supports our finding that the number of consultations is related to the odds of prescribing pain medications. We found that the odds of prescribing any pain medication are significantly higher if the physicians did not self-dispense drugs. This finding is surprising as studies report that the status of self-dispensing is associated with increased frequencies of drug prescribing [32].

### 4.4. Co-Medications and LBP

One third of the patients, 36%, received co-medications: proton-pump-inhibitors and muscle relaxants were the most commonly used. Co-medication use, especially the use of muscle relaxants or antidepressants, is common among patients with LBP and their use is mentioned in many guidelines [10,26]. In contrast to the US, the use of both muscle relaxants and antidepressants is less common in our cohort [11,17]. Muscle relaxants are used twice as much as antidepressants, according to the current evidence favoring muscle relaxants over antidepressants [26,33], for which evidence is limited [34]. In contrast to muscle relaxants or antidepressants, sleeping pills are not mentioned in the guidelines. The association between pain and sleep disturbances is well described [35] and one study reported that, approximately, two-thirds of the patient with LBP suffers from sleeping disturbances [36]. However, sleeping pills were rarely prescribed in our cohort: we found a low rate of PPI prescriptions in patients with concurrent NSAID therapy. PPI are recommended in many guidelines to prevent NSAID associated gastrointestinal bleedings [37] but data on the dose and duration of NSAID treatments would be necessary to finally judge the appropriateness of this finding.

### 4.5. Limitations and Strengths

The study has some limitations. First, we cannot exclude specific pain medication prescriptions for a reason other than an acute LBP episode (i.e., alternative pain, or prescription of pain medications as a fever-reducing medication). This is because ATC codes do not depend on ICPC-2 code in FIRE database. Therefore, our approach was conservative and all patients with ICPC-2 diagnosis codes, qualifying for a pain medication beside the LBP diagnosis, (i.e., all cancer or trauma diagnosis) were excluded. Moreover, a long baseline period of twelve months minimized the chance of missing a diagnosis qualifying for a pain medication. Second, we could only analyze the first LBP episode for each patient. Due to the different structures of medical software used by conducting GPs, we could not definitely exclude cases where diagnosis codes were reported along the subsequent patient consultations, even if the reason of encounter was for other complaints. Finally, we could not exclude the possibility that patients take additional OTC pain medications, which are not prescribed by the GP. Therefore, there is a possibility that we overestimate the number of patients without any pain medication and underestimate the number of pain medication taken.

On the other hand, the main strength of this study is the large sample size of patients. The validity of our data and consequently of our results, is supported by the fact that the distribution of age, gender as well as the mean of the number of consultations and the number of patients receiving any kind of pain medication, are in line with the numbers reported in similar health care settings [9,38,39] and available national data [4].

## 5. Conclusions

The study described patterns of pain medication therapy in a large cohort in Swiss primary care. More than one-third of the patients had no pain medication and 86% of the pain medications was at least a NSAID, in line with current guidelines. However, the still substantial use of opioids suggests that GPs have to be aware of handling of opioids in non-cancer pain.

## Figures and Tables

**Table 1:** Basic characteristics of 10,331 patients with the diagnosis of low back pain.

Variables	All Patients	Back Syndrome with Radiating Pain	Back Syndrome without Radiating Pain	Low Back Symptom/Complaint	Combination of Symptoms ^1^	*p*
Number of patients, *n*	10,331	1758	2465	5663	445	
Number of consultations per patient, mean (SD^2^; range (min–max))	2.12 (3.25; (1–63))	2.58 (3.74; (1–43))	1.89 (2.74; (1–41))	2.01 (3.25; (1–63))	2.83 (3.53; (1–28))	<0.001
Age, mean (SD, range (min–max))	51.66 (18.24; (18–100))	56.52 (17.25; (18–97))	49.38 (18.32; (18–97))	51.01 (18.31; (18–100))	53.41 (17.14; (18–93))	0.005
Sex						<0.001
male, *n* (%)	5042 (48.8)	835 (47.5)	1271 (51.6)	2709 (47.8)	227 (51.0)	
female, *n* (%)	5289 (51.2)	923 (52.5)	1194 (48.4)	2954 (52.2)	218 (49.0)	

^1^ Of the 445 patients with combinations, there were 94 patients with combinations of back syndrome without radiating pain and with symptom/complaint. ^2^ SD: standard deviation. Bold: Significant results are presented in bold.

**Table 2:** Pain medication prescriptions in 10,331 patients with a low back pain diagnosis.

Variables	Total	Back Syndrome with Radiating Pain	Back Syndrome without Radiating Pain	Low Back Symptom/Complaint	Combination of Symptoms	*p*
	*n*^1^ = 10,331	*n* = 1758	*n* = 2465	*n* = 5663	*n* = 445	
**A—Basic prescribing patterns ^2^**	***n* (%)**	***n* (%)**	***n* (%)**	***n* (%)**	***n* (%)**	
Patients without any pain medication	3882 (37.6)	588 (33.4)	814 (33.0)	2360 (41.7)	120 (27.0)	**<0.001**
Patients with at least one pain medication	6449 (62.4)	1170 (66.6)	1651 (67.0)	3303 (58.3)	325 (73.0)	**<0.001**
Patients with one pain medication	3826 (37.0)	614 (34.9)	1071 (75.7)	1992 (35.2)	149 (33.5)	**<0.001**
Patients with two or more pain medications	2623 (25.4)	556 (31.6)	580 (23.5)	1311 (23.2)	176 (39.6)	**<0.001**
Patients in which the first prescribed pain medication after or at diagnosis was						
NSAID	2813 (27.2)	465 (26.5)	813 (33.0)	1408 (24.9)	127 (28.5)	**<0.001**
Opioid	505 (4.9)	132 (7.5)	104 (4.2)	221 (3.9)	48 (10.8)	**<0.001**
Paracetamol	853 (8.3)	118 (6.7)	153 (6.2)	528 (9.3)	54 (12.1)	**<0.001**
**B—Specific prescribing patterns ^3^**						
NSAID						
Patients receiving NSAIDs	5545 (86.0)	1014 (86.7)	1478 (89.5)	2779 (84.1)	274 (84.3)	**<0.001**
Patients receiving only NSAIDs	3088 (47.9)	514 (43.9)	966 (58.5)	1509 (45.7)	99 (30.5)	**<0.001**
Opioids						
Patients receiving opioids	1417 (22.0)	367 (31.4)	278 (16.8)	652 (19.7)	120 (36.9)	**<0.001**
Patients receiving only opioids	178 (2.8)	44 (3.8)	38 (2.3)	81 (2.5)	15 (4.6)	**0.033**
Paracetamol						
Patients receiving paracetamol	2544 (39.4)	441 (37.7)	512 (31.0)	1439 (43.6)	152 (46.8)	**<0.001**
Patients receiving only paracetamol	602 (9.3)	89 (7.6)	115 (7.0)	372 (11.3)	26 (8.0)	**<0.001**
Combination therapies						
Patients receiving NSAIDs and paracetamol	1342 (20.8)	200 (17.1)	292 (17.7)	770 (23.3)	80 (24.6)	**<0.001**
Patients receiving NSAIDs and opioids	639 (9.9)	171 (14.6)	135 (8.2)	274 (8.3)	59 (18.2)	**<0.001**
Patients receiving NSAIDs, opioids and paracetamol	476 (7.4)	129 (11.0)	85 (5.1)	226 (6.8)	36 (11.1)	**<0.001**
Patients receiving opioids and paracetamol	124 (1.9)	23 (2.0)	20 (1.2)	71 (2.1)	10 (3.1)	0.068
**C—Timing ^4^**						
Time from diagnosis to first NSAID prescription	8.71 (30.11)	8.38 (28.82)	8.53 (30.59)	8.76 (29.95)	10.41 (33.59)	0.966
Time from diagnosis to first Opioid prescription	7.53 (25.14)	6.61 (21.95)	7.51 (26.45)	6.24 (23.11)	16.04 (36.31)	0.903
Time from diagnosis to first paracetamol prescription	13.13 (37.13)	11.78 (33.68)	14.80 (36.27)	13.42 (38.84)	8.56 (29.23)	0.807

^1^*n* = number of patients; NSAIDs: non-steroidal anti-inflammatory drugs. ^2^ Table part A shows basic prescribing patterns. Values are presented as absolute numbers and percentage. Percentage values refer to the overall patients analyzed. ^3^ Table part B shows specific prescribing patterns of the drug classes of interest. Values are presented as absolute numbers and percentage. Percentages values refer to the amount of patients receiving any drug. Combination therapies with tramadol and paracetamol are counted only once in the opioid group. ^4^ Table part C shows time (in days) from diagnosis to first prescription in patient groups in which the pain medication was used as first line therapy. Values are presented as mean (SD). Bold: Significant results are presented in bold.

**Table 3:** Association between patient and GP characteristics (predictors) and pain medication prescription (binary outcome): mixed-effects logistic regression analysis, accounting for correlation within GP.

Outcome	Prescription of Any Pain Medication			NSAID		Opioid		Paracetamol	
Predictor (Reference, Where Applicable)	Number of Patients/GP	OR (95% CI)	*p*	OR (95% CI)	*p*	OR (95% CI)	*p*	OR (95% CI)	*p*
**GP characteristics**									
Age ^1^ (continuous)	*n* = 9825, GP = 161	0.99 (0.98, 1.01)	0.195			0.98 (0.96, 0.99)	**0.007**		
						0.97 (0.95, 0.99)	**<0.001**		
Age (age ≤ 55)				1.07 (0.90, 1.26)	0.450			1.00 (0.83, 1.20)	0.988
								0.84 (0.66, 1.06)	0.132
Male gender (female)	*n* = 10252, GP = 178	1.67 (1.11, 2.5)	**0.014**	1.86 (1.25, 2.77)	**0.002**	1.38 (0.92, 2.07)	0.118	1.27 (0.88, 1.85)	0.206
				1.32 (0.92, 1.90)	0.130				
Type of practice (single practice) Double practice	*n* = 10331, GP = 180	1.13 (0.40, 3.19)	0.820	1.27 (0.46, 3.51)	0.647	1.35 (0.54, 3.34)	0.521	1.10 (0.45, 2.65)	0.838
									
Group practice		1.25 (0.67, 2.35)	0.487	1.19 (0.64, 2.20)	0.579	1.00 (0.57, 1.76)	1.000	1.12 (0.66, 1.92)	0.668
									
Years in practice	*n* = 9731, GP = 148	0.99 (0.98, 1.01)	0.431	0.99 (0.98, 1.01)	0.882	0.98 (0.97, 1.00)	0.050	0.99 (0.97, 1.00)	0.077
								**0.98 (0.96, 1.00)**	**0.012**
Self-Employed (employee)	*n* = 9978, GP = 161	3.03 (2.04, 4.51)	**<0.001**	3.13 (2.13, 4.60)	**<0.001**	2.59 (1.67, 4.03)	**<0.001**	1.71 (1.16, 2.52)	**0.007**
		2.36 (1.64, 3.40)	**<0.001**	2.38 (1.63, 3.47)	**<0.001**	2.34 (1.50, 3.63)	**<0.001**	1.56 (1.07, 2.28)	**0.020**
Employment level (100%) <50%	*n* = 8938, GP = 143	0.41 (0.20, 0.84)	**0.016**	0.52 (0.26, 1.06)	0.073	0.42 (0.19, 0.91)	**0.028**	0.57 (0.29, 1.11)	0.098
									
50–79%		0.55 (0.34, 0.87)	**0.010**	0.56 (0.36, 0.88)	**0.013**	0.59 (0.37, 0.94)	**0.025**	0.72 (0.48, 1.08)	0.113
									
80–99%		0.82 (0.51, 1.34)	0.437	0.90 (0.56, 1.44)	0.666	0.70 (0.44, 1.11)	0.132	0.95 (0.63, 1.45)	0.822
									
Self-dispensing (yes) No	*n* = 10311, GP = 175	1.98 (1.29, 3.05)	**0.002**	2.00 (1.32, 3.04)	**0.001**	1.21 (0.83, 1.78)	0.315	1.44 (0.97, 2.14)	0.070
		1.76 (1.22, 2.52)	**0.002**	1.79 (1.26, 2.55)	**0.001**				
**Patient characteristics**									
Age at diagnosis > 50 (≤50) years old	*n* = 10331, GP = 180	0.83 (0.76, 0.91)	**<0.001**	1.11 (1.01, 1.21)	**0.024**	0.52 (0.46, 0.59)	**<0.001**	0.72 (0.65, 0.79)	**<0.001**
				1.24 (1.13, 1.36)	**<0.001**	0.66 (0.58, 0.76)	**<0.001**	0.84 (0.75, 0.93)	**0.001**
Male gender (female)	*n* = 10331, GP = 180	1.07 (0.97, 1.16)	0.167	1.17 (1.08, 1.28)	**<0.001**	0.99 (0.88, 1.12)	0.912	0.79 (0.72, 0.87)	**<0.001**
		1.10 (1.00, 1.21)	**0.044**	1.20 (1.09, 1.31)	**<0.001**			0.82 (0.74, 0.90)	<0.001
Number of consultations after diagnosis	*n* = 10331, GP = 180	2.21 (2.05 2.39)	**<0.001**	1.24 (1.2 1.27)	**<0.001**	1.24 (1.22, 1.27)	**<0.001**	1.24 (1.21, 1.27)	**<0.001**
		2.20 (2.04, 2.37)	**<0.001**	1.27 (1.23, 1.31)	**<0.001**	1.27 (1.24, 1.30)	**<0.001**	1.25 (1.22, 1.28)	**<0.001**
Diagnosis Code ^2^ (Back syndrome with radiating pain) Low back symptom/complaint	*n* = 9886, GP = 179	0.88 (0.76, 1.01)	0.065	0.92 (0.81, 1.06)	0.257	0.92 (0.81, 1.06)	0.257	0.81 (0.70, 0.94)	**0.005**
									
Back syndrome without radiating pain		1 (0.86, 1.17)	0.964	1.03 (0.89, 1.19)	0.726	1.03 (0.89, 1.19)	0.726	0.86 (0.74, 1.02)	0.076
									

Results for the univariable analysis were in the corresponding upper row and results for multivariable analysis in the corresponding lower row (grey shaded). If the predictor was not considered in multivariable analysis, the fields are blank. For multivariable analysis, the following numbers apply depending on the endpoint: 1: All prescriptions: *n* = 9958, GP = 156, ICC = 0.14, 2: NSAID prescriptions: N = 9958, GP = 156, ICC = 0.13; 3: Opioids: *n* = 9798, GP = 156, ICC = 0.13; 4: Paracetamol: *n* = 9696, GP = 146, ICC = 0.11. GP: general practitioner; OR: odds ratio; CI: confidence interval; *n*: number of patients; ICC: intra-class correlation coefficient. ^1^ Age of GP was defined as continuous or binary variable according to computational requirements. ^2^ Excluding patients with combined diagnosis. Bold: Significant results are presented in bold.

## Data Availability

The data presented in this study are available on request from the corresponding author.

## Supplementary Materials

The following are available online at https://www.mdpi.com/article/10.3390/jcm10071366/s1, Table S1: Detail of pain medication prescriptions in 10,331 patients with a low back pain diagnosis, Table S2: Relative number of prescriptions per patient stratified by the time of prescription and by diagnosis group (6449 patients), Table S3: Time of first pain medication prescriptions in 6449 patients with low back pain, Table S4: Time patterns (in days) of pain medication prescription in 6449 patients with a low back pain diagnosis, Table S5: Counts of prescriptions (outcome) per patient stratified by LBP groups. Poisson regression models (multivariable analysis) corrected for repeated measurements within patients, Table S6: Counts of prescriptions (outcome) per patient stratified by LBP groups. Poisson regression models (multivariable analysis) corrected for repeated measurements within patients, Table S7: Co-medications in 10,331 patients with a low back pain diagnosis, List of all used ATC codes, List of all ICPC codes excluded.
