# Supplementary material for: Prescribing Patterns of Pain Medications in Unspecific Low Back Pain in Primary Care: A Retrospective Analysis
Source: J Clin Med. 2021 Mar 26;10(7):1366. doi: 10.3390/jcm10071366 (PMC8036853; doi:10.3390/jcm10071366)
Supplement: Supplementary file 1 [file jcm-10-01366-s001.pdf]

Supplementary Material, Table S1: Detail of pain medication prescriptions in 10,331 patients with a low back pain diagnosis

	**Total**	**Back syndrome with radiating pain**	**Back syndrome without radiating pain**	**Low back symptom / complaint**	**Combination of symptoms**	***p***
	N = 10,331	*N* = 1758	*N* = 2465	*N* = 5663	*N* = 445	
**NSAID** [1]						
Absolute NSAIDs prescriptions[2]						
Diclofenac	2502 (45.1)	482 (47.5)	700 (47.4)	1168 (42.0)	152 (55.5)	**0.001**
Etodolac	67 (1.2)	26 (2.6)	10 (0.7)	24 (0.9)	7 (2.6)	**<0.001**
Ketorolac	9 (02)	2 (0.2)	1 (0.1)	6 (0.2)	0 (0.0)	0.554
Diclofenac combinations	76 (1.4)	20 (2.0)	17 (1.2)	36 (1.3)	3 (1.1)	0.187
Ibuprofen	2208 (39.8)	368 (36.3)	558 (37.8)	1181 (42.5)	101 (36.9)	**<0.001**
Naproxen	254 (4.6)	77 (7.6)	116 (7.8)	47 (1.7)	14 (5.1)	**<0.001**
Naproxen/Esomeprazol	279 (5.0)	40 (3.9)	56 (3.8)	167 (6.0)	16 (5.8)	**0.002**
Mefenamic acid	599 (10.8)	86 (8.5)	149 (10.1)	338 (12.2)	26 (9.5)	**0.003**
Celecoxib	89 (1.6)	16 (1.6)	21 (1.4)	50 (1.8)	2 (0.7)	0.641
Diclofenac topical	1638 (29.5)	324 (32.0)	433 (29.3)	822 (29.6)	59 (21.5)	0.289
**Opioids**						
Absolute opioid prescriptions[2]						
Morphine	63 (4.4)	13 (3.5)	13 (4.7)	32 (4.9)	5 (4.2)	0.617
Hydromorphone	1 (0.1)	0 (0.0)	0 (0.0)	1 (0.2)	0 (0.0)	1.000
Oxycodone	87 (6.1)	25 (6.8)	19 (6.8)	41 (6.3)	2 (1.7)	0.926
Oxycodone/Naloxon	157 (11.1)	38 (10.4)	22 (7.9)	84 (12.9)	13 (10.8)	0.066
Pethidine	1 (0.1)	1 (0.3)	0 (0.0)	0 (0.0)	0 (0.0)	0.497
Fentanyl	58 (4.1)	18 (4.9)	12 (4.3)	26 (4.0)	2 (1.7)	0.756
Buprenorphine	12 (0.8)	1 (0.3)	5 (1.8)	5 (0.8)	1 (0.8)	0.084
Tramadol and paracetamol[3]	136 (9.6)	30 (8.2)	25 (9.0)	72 (11.0)	9 (7.5)	**0.278**
Tilidine	19 (1.3)	1 (0.3)	4 (1.4)	13 (2.0)	1 (0.8)	**0.078**
Tramadol	1044 (73.7)	273 (74.4)	217 (78.1)	457 (70.1)	97 (80.8)	**0.033**
**Combination therapies**						

Most common combinations						
Diclofenac systemic and topical	373 (14.2)	95 (17.1)	106 (18.3)	158 (12.1)	14 (8.0)	**<0.001**
Diclofenac + Tramadol	395 (15.1)	117 (21.0)	88 (15.2)	137 (10.5)	53 (30.1)	**<0.001**
Ibuprofen + topical Diclofenac	359 (13.7)	70 (12.6)	96 (16.6)	183 (14.0)	10 (5.7)	0.145

[1] N= number of patients; NSAIDs: non-steroidal anti-inflammatory drugs. Bold: Significant results are presented in bold.
[2] percentage value refers to the amount of patients (receiving a drug of this class), in which the specific drug is prescribed.
[3] Combination therapies with tramadol and paracetamol are counted only once in the opioid group.

Supplementary Material, Table S2: Relative number of prescriptions per patient stratified by the time of prescription and by diagnosis group (6`449 patients).

Number of prescriptions per patient	Total	Back syndrome with radiating pain	Back syndrome without radiating pain	Low back symptom / complaint	Combination of symptoms	*p*
**Overall[1]**	1.84 (1.01)<br>*N* = 6449	1.96 (1.04)<br>*N* = 1170	1.75 (0.96)<br>*N* = 1651	1.82 (1.02)<br>*N* = 3303	2.04 (1.04)<br>*N* = 325	**<0.001**
Before diagnosis	1.59 (0.88)	1.66 (0.91)	1.58 (0.86)	1.57 (0.88)	1.54 (0.78)	0.082
At diagnosis	1.45 (0.72)	1.51 (0.70)	1.38 (0.64)	1.47 (0.77)	1.43 (0.64)	**<0.001**
Within week 1 after diagnosis	1.68 (0.94)	1.65 (0.83)	1.58 (0.80)	1.74 (1.07)	1.67 (0.70)	0.096
Within week 2-4 after diagnosis	1.61 (0.92)	1.54 (0.77)	1.56 (0.88)	1.65 (1.03)	1.64 (0.76)	0.132
Later than four weeks after diagnosis	1.61 (0.94)	1.59 (0.83)	1.55 (0.86)	1.63 (1.03)	1.70 (0.83)	0.271
**NSAIDS**	1.39 (0.65)<br>*N* = 5545	1.42 (0.64)<br>*N* = 1014	1.39 (0.64)<br>*N* = 1478	1.38 (0.67)<br>*N* = 2779	1.39 (0.62)<br>*N* = 274	0.254
Before diagnosis	1.31 (0.61)	1.33 (0.57)	1.34 (0.60)	1.30 (0.63)	1.21 (0.43)	0.441
At diagnosis	1.19 (0.47)	1.21 (0.45)	1.20 (0.44)	1.19 (0.51)	1.12 (0.35)	0.488
Within week 1 after diagnosis	1.25 (0.62)	1.16 (0.44)	1.19 (0.49)	1.33 (0.76)	1.14 (0.35)	**0.003**
Within week 2-4 after diagnosis	1.24 (0.61)	1.18 (0.45)	1.24 (0.55)	1.29 (0.73)	1.14 (0.35)	0.099
Later than four weeks after diagnosis	1.28 (0.62)	1.25 (0.48)	1.28 (0.56)	1.31 (0.71)	1.18 (0.43)	0.244
**Opioids**	1.13 (0.41)<br>*N* = 1417	1.13 (0.40)<br>*N* = 367	1.14 (0.41)<br>*N* = 278	1.14 (0.43)<br>*N* = 652	1.09 (0.29)<br>*N* = 120	0.790
Before diagnosis	1.11 (0.34)	1.14 (0.37)	1.14 (0.35)	1.09 (0.33)	1.10 (0.31)	0.440
At diagnosis	1.03 (0.16)	1.03 (0.17)	1.03 (0.17)	1.03 (0.16)	1.02 (0.13)	0.971
Within week 1 after diagnosis	1.04 (0.20)	1.06 (0.24)	1.01 (0.12)	1.04 (0.21)	1.03 (0.17)	0.305
Within week 2-4 after diagnosis	1.08 (0.33)	1.05 (0.21)	1.09 (0.28)	1.13 (0.43)	1.00 (0.00)	0.193
Later than four weeks after diagnosis	1.14 (0.41)	1.15 (0.42)	1.19 (0.42)	1.15 (0.45)	1.02 (0.12)	0.717

[1]: Including non-steroidal anti-inflammatory drug (NSAID), opioids, and paracetamol. Data are reported as mean (SD). Bold: Significant results are presented in bold.

Supplementary Material, Table S3: Time of first pain medication prescriptions in 6,449 patients with low back pain.

Time of first pain medication prescription	Total	Back syndrome with radiating pain	Back syndrome without radiating pain	Low back symptom / complaint	Combination of symptoms	*p*
**Overall**	*N* = 6449	*N* = 1170	*N* = 1651	*N* = 3303	*N* = 325	
Before diagnosis	3003 (46.6)	600 (51.3)	712 (43.1)	1546 (46.8)	145 (44.6)	**<0.001**
At diagnosis	2899 (45.0)	466 (39.8)	820 (49.7)	1464 (44.3)	149 (45.8)	**<0.001**
Within week 1 after diagnosis	117 (1.8)	23 (2.0)	17 (1.0)	70 (2.1)	7 (2.2)	**0.022**
Within week 2-4 after diagnosis	97 (1.5)	29 (2.5)	9 (0.5)	52 (1.6)	7 (2.2)	**<0.001**
Later than four weeks after diagnosis	333 (5.2)	52 (4.4)	93 (5.6)	171 (5.2)	17 (5.2)	0.371
**NSAIDs**	*N* = 5545	*N* = 1014	*N* = 1478	*N* = 2779	*N* = 274	
Before diagnosis	2402 (43.3)	488 (48.1)	583 (39.4)	1217 (43.8)	114 (41.6)	**<0.001**
At diagnosis	2622 (47.3)	429 (42.3)	783 (53.0)	1289 (46.4)	121 (44.2)	**<0.001**
Within week 1 after diagnosis	116 (2.1)	14 (1.4)	26 (1.8)	67 (2.4)	9 (3.3)	0.094
Within week 2-4 after diagnosis	87 (1.6)	26 (2.6)	8 (0.5)	47 (1.7)	6 (2.2)	**<0.001**
Later than four weeks after diagnosis	318 (5.7)	57 (5.6)	78 (5.3)	159 (5.7)	24 (8.8)	0.833
**Paracetamol**	*N* = 2544	*N* = 441	*N* = 512	*N* = 1439	*N* = 152	
Before diagnosis	1221 (48.0)	239 (54.2)	263 (51.4)	656 (45.6)	63 (41.4)	**0.002**
At diagnosis	896 (35.2)	119 (27.0)	162 (31.6)	561 (39.0)	54 (35.5)	**<0.001**
Within week 1 after diagnosis	73 (2.9)	22 (5.0)	8 (1.6)	37 (2.6)	6 (3.9)	**0.004**
Within week 2-4 after diagnosis	74 (2.9)	16 (3.6)	11 (2.1)	37 (2.6)	10 (6.6)	0.342
Later than four weeks after diagnosis	280 (11.0)	45 (10.2)	68 (13.3)	148 (10.3)	19 (12.5)	0.152
**Opioids**	*N* = 1417	*N* = 367	*N* = 278	*N* = 652	*N* = 120	
Before diagnosis	362 (25.5)	96 (26.2)	70 (25.2)	176 (27.0)	20 (16.7)	0.843
At diagnosis	634 (44.7)	158 (43.1)	137 (49.3)	287 (44.0)	52 (43.2)	0.238
Within week 1 after diagnosis	137 (9.7)	39 (10.6)	22 (7.9)	64 (9.8)	12 (10.0)	0.500
Within week 2-4 after diagnosis	93 (6.6)	33 (9.0)	10 (3.6)	43 (6.6)	7 (5.8)	**0.024**
Later than four weeks after diagnosis	191 (13.5)	41 (11.2)	39 (14.0)	82 (12.6)	29 (24.2)	0.551

Bold: Significant results are presented in bold.

Supplementary Material, Table S4: Time patterns (in days) of pain medication prescription in 6,449 patients with a low back pain diagnosis

	Total	Back syndrome with radiating pain	Back syndrome without radiating pain	Low back symptom/complaint	Combination of symptoms	*p*
Patients with **more than one NSAID** (after or at diagnosis)	*N* = 365	*N* = 68	*N* = 119	*N* = 165	*N* = 13	
Time from the day of diagnosis to the day of the first therapy	5.44 (23.14) (0-170)	4.88 (21.09) (0-136)	3.58 (18.80) (0-137)	7.43 (27.27) (0-170)	0.00 (0.00) (0-0)	0.381
Time delay between first and second (different) NSAID prescription	24.20 (46.06) (0-180)	18.25 (39.08) (0-168)	17.93 (41.43) (0-163)	28.41 (49.07) (0-180)	59.31 (62.16) (0-172)	0.097
Cases **with NSAIDs and paracetamol** (after or at diagnosis)	*N* = 457	*N* = 56	*N* = 80	*N* = 288	*N* = 33	
Time from the day of diagnosis to the day of the first NSAID therapy Mean (SD)	11.04 (34.07) (0-176)	8.43 (24.77) (0-121)	5.17 (21.71) (0-130)	11.43 (36.82) (0-176)	26.33 (42.69) (0-139)	0.308
Time delay (in days) between first NSAID prescription and Paracetamol prescription	14.37 (35.86) (0-179)<br>*N* = 414	13.72 (34.34) (0-144)<br>N=50	18.24 (40.12) (0-179)<br>*N* = 75	12.45 (33.79) (0-166)<br>*N* = 265	24.83 (45.71) (0-141)<br>*N* = 24	0.454
Time from the day of diagnosis to the day of the first Paracetamol therapy Mean (SD)	22.88 (44.87) (0-153)	0.00 (0.00) (0-0)	36.20 (51.65) (0-125)	34.48 (53.29) (0-153)	1.11 (2.26) (0-6)	0.295
Time delay (in days) between Paracetamol prescription and first NSAID prescription	48.98 (48.53) (0-172)<br>*N* = 43	62.50 (49.26) (0-121)<br>*N* = 6	7.20 (3.35) (5-13)<br>*N* = 5	45.48 (48.57) (0-172)<br>N=23	72.11 (49.90) (0-139)<br>*N* = 9	0.135
Cases with **NSAIDs and Opioid** (after or at diagnosis)	*N* = 370	*N* = 97	*N* = 75	*N* = 160	*N* = 38	
Time from the day of diagnosis to the day of starting the first NSAID therapy	6.96 (24.03) (0-165)	6.92 (23.71) (0-142)	8.97 (34.58) (0-165)	4.85 (16.22) (0-87)	12.00 (26.94) (0-91)	0.446<br><br>0.304

Time delay between first NSAID prescription and opioid prescription	9.07 (27.19) (0-177)<br>*N* = 346	4.87 (14.21) (0-96)<br>*N* = 92	9.71 (29.10) (0-151)<br>*N* = 69	9.93 (29.84) (0-177)<br>*N* = 151	15.29 (35.86) (0-151)<br>*N* = 34	
Time from the day of diagnosis to the day of starting the first Opioid therapy	3.04 (8.88) (0-42)	2.00 (2.83) (0-6)	7.00 (17.15) (0-42)	2.33 (4.61) (0-13)	0.00 (0.00) (0-0)	0.621
Time delay between opioid prescription and first NSAID prescription	46.79 (46.40) (1-163)<br>*N* = 24	89.80 (28.34) (63-138)<br>*N* = 5	55.00 (70.45) (1-163)<br>*N* = 6	25.89 (25.62) (2-76)<br>*N* = 9	27.75 (20.02) (3-45)<br>*N* = 4	0.057
Cases with **paracetamol and Opioid** (after or at diagnosis)[1]	*N* = 53	*N* = 14	*N* = 9	*N* = 24	*N* = 6	
Time from the day of diagnosis to the day of starting the first opioid therapy	23.57 (43.95) (0-153)	15.64 (35.11) (0-119)	9.22 (18.68) (0-49)	26.04 (46.84) (0-153)	53.67 (67.61) (0-148)	0.505
Time delay between first opioid prescription and paracetamol prescription	15.51 (43.12) (0-178)<br>*N* = 39	11.82 (38.54) (0-128)<br>*N* = 11	0.00 (0.00) (0-0)<br>*N* = 8	18.56 (42.00) (0-133)<br>*N* = 16	44.50 (89.00) (0-178)<br>*N* = 4	0.498
Time from the day of diagnosis to the day of starting the first paracetamol therapy	15.36 (41.32) (0-42)	0.00 (0.00) (0-0)	0.00 (0.00) (0-0)	9.12 (25.81) (0-73)	71.00 (100.41) (0-142)	-
Time delay between paracetamol prescription and first opioid prescription	49.93 (44.11) (1-135)<br>*N* = 14	73.00 (41.61) (38-119)<br>*N* = 3	49.00<br>*N* = 1	49.38 (50.58) (1-135)<br>*N* = 8	18.00 (16.97) (6-30)<br>*N* = 2	-

[1]: We considered the first NSAID and the first opioid therapy only

Mean values, standard deviation and range (min-max) are reported, even if the distribution is skewed. In fact, mean was chosen as it includes every value. Instead, median lacks the representatives of data as it only selects the positional middle value.

Supplementary Material, Table S5: Counts of prescriptions (outcome) per patient stratified by LBP groups. Poisson regression models (multivariable analysis) corrected for repeated measurements within patients.

	**All patients** *N* = 22,968, Patients=6124		**Back syndrome with radiating pain** *N* = 5260, Patients=1170		**Back syndrome without radiating pain** *N* = 5510, Patients=1651		**Low back symptom / complaint** *N* = 12,198, Patients=3303	
**Predictor (reference, where applicable)**	RR[1] (95% CI)	*p*	RR (95% CI)	*p*	RR (95% CI)	*p*	RR (95% CI)	*p*
Time of pain medication								
Before diagnosis (at diagnosis)	1.02 (0.99, 1.05)	0.255	1.00 (0.94, 1.07)	0.889	1.02 (0.97, 1.09)	0.413	1.02 (0.97, 1.06)	0.474
0 - 2 months after diagnosis (at diagnosis)	1.02 (0.97, 1.08)	0.458	0.96 (0.87, 1.07)	0.504	1.03 (0.92, 1.16)	0.594	1.04 (0.97, 1.11)	0.314
2 – 6 months after diagnosis (at diagnosis)	1.04 (1.00, 1.08)	**0.025**	0.98 (0.92, 1.06)	0.667	1.04 (0.97, 1.12)	0.234	1.06 (1.01, 1.11)	**0.017**
Pain Medication								
Opioids (NSAIDS)	0.86 (0.83, 0.89)	**<0.001**	0.90 (0.83, 0.96)	**0.004**	0.86 (0.79, 0.93)	**<0.001**	0.84 (0.79, 0.88)	**<0.001**
Paracetamol (NSAIDS)	0.79 (0.77, 0.82)	**<0.001**	0.82 (0.77, 0.87)	**<0.001**	0.80 (0.75, 0.85)	**<0.001**	0.78 (0.75, 0.81)	**<0.001**
Diagnosis Code[2]								
Low back symptom / complaint (Back syndrome with radiating pain)	1.02 (0.98, 1.05)	0.316						
Back syndrome without radiating pain (Back syndrome with radiating pain)	1.01 (0.97, 1.04)	0.723						

[1] RR = risk ratio; CI (Confidence Intervals). Bold: Significant results are presented in bold.
[2] Excluding patients with combined diagnosis.

Supplementary Material, Table S6: Association between patient and GP characteristics (predictors) and pain medication prescription (binary outcome). Mixed-effects logistic regression analysis, accounting for correlation within GP for all patients, for all patients and by diagnosis groups.

Prescription of any pain medication												
	**All Patients**			**Patients with Back syndrome with radiating pain**			**Patients with Back syndrome without radiating pain**			**Patients with Low back symptom / complaint**		
**Predictor (reference, where applicable)**	*N*/GP	OR (95% CI)	*p*	*N* / GP	OR (95% CI)	*p*	*N*/GP	OR (95% CI)	*p*	*N*/GP	OR (95% CI)	*p*
**GP characteristics**												
Age	9394/160	0.99 (0.97, 1)	0.157	1706 / 114	1.01 (0.983 , 1.04)	0.417	2400/116	0.994 (0.97, 1.02)	0.598	5288/133	0.99 (0.97, 1.01)	0.281
Male gender (female)	9808/177	1.62 (1.08 , 2.43)	**0.020**	1749 / 126	1.31 (0.69 , 2.5)	0.407	2455/126	2.23 (1.31 , 3.79)	**0.003**	5604/147	1.78 (1.14 , 2.8)	**0.012**
Type of practice double (single practice)	9886/179	1.16 (0.412 , 3.26)	0.779	1758 / 128	1.41 (0.347 , 5.77)	0.629	2465/128	1.15 (0.363 , 3.66)	0.81	5663/149	0.96 (0.24, 3.85)	0.948
Group practice (single practoce)	9886/179	1.29 (0.69 , 2.4)	0.429	1758 / 128	1.45 (0.596 , 3.53)	0.414	2465/128	1.34 (0.61 , 2.93)	0.468	5663/149	1.16 (0.57 , 2.34)	0.686
Years in practice	9300/147	0.99 (0.98 , 1.01)	0.341	1686 / 108	1.01 (0.982 , 1.03)	0.579	2374/110	0.994 (0.973 , 1.02)	0.6	5240/123	0.997 (0.98, 1.01)	0.713
Self-Employed (employee)	9545/160	2.91 (1.96 , 4.31)	**<0.001**	1714 /114	4.44 (2.38 , 8.29)	**<0.001**	2409/118	2.94 (1.73 , 4.99)	**<0.001**	5422/134	3.48 (2.29 , 5.28)	**<0.001**
	**9525/155**	**2.28 (1.59, 3.28)**	**<0.001**	**1707/112**	**3.09 (1.67, 5.73)**	**<0.001**	**2397/115**	**1.79 (1.10, 2.91)**	**0.019**	**5421/133**	**2.86 (1.93, 4.23)**	**<0.001**
Self-dispensing No (yes)	9866/174	1.99 (1.3 , 3.05)	**0.002**	1751 / 126	2.09 (1.14 , 3.86)	**0.018**	2453/125	3.12 (1.91 , 5.08)	**<0.001**	5662/148	1.69 (1.03, 2.76)	**0.037**
	**9525/155**	**1.74 (1.21, 2.49)**	**0.003**	**1707/112**	**1.44 (0.82, 2.54)**	0.206	**2397/115**	**2.58 (1.68, 3.96)**	**<0.001**	**5421/133**	**1.48 (1.00, 2.21)**	0.053
Employment level (100%) <50% (100%)	8557/142	0.42 (0.20 , 0.86)	**0.018**	1471 / 100	0.59 (0.196 , 1.8)	0.358	2089/105	0.371 (0.15 , 0.90)	**0.029**	4997/118	0.42 (0.19 , 0.93)	**0.033**
50-79% (100%)	8557/142	0.56 (0.35 , 0.88)	**0.013**	1471 / 100	0.51 (0.256 , 1)	0.051	2089/105	0.584 (0.33 , 1.03)	0.065	4997/118	0.50 (0.30 , 0.83)	**0.008**
80-99% (100%)	8557/142	0.84 (0.52, 1.36)	0.471	1471 / 100	1.4 (0.641 , 3.05)	0.4	2089/105	0.751 (0.40 , 1.39)	0.364	4997/118	0.90 (0.54, 1.51)	0.693

**Patient characteristics**												
Number of consultations after diagnosis	9886/179	2.18 (2.02 , 2.35)	**<0.001**	1758 / 128	2.58 (2.15 , 3.08)	**<0.001**	2465/128	2.24 (1.9 , 2.64)	**<0.001**	5663/149	2.09 (1.89, 2.31)	**<0.001**
	**9525/155**	**2.16 (2.00, 2.34)**	**<0.001**	**1707/112**	**2.58 (2.15, 3.10)**	**<0.001**	**2397/115**	**2.19 (1.86, 2.59)**	**<0.001**	**5421/133**	**2.07 (1.87, 2.29)**	**<0.001**
Age at diagnosis >50 (<=50) years old	9886/179	0.817 (0.74 , 0.90)	**<0.001**	1758 / 128	0.91 (0.723 , 1.15)	0.451	2465/128	0.671 (0.56 , 0.81)	**<0.001**	5663/149	0.84 (0.74 , 0.95)	**0.004**
Male gender (female)	9886/179	1.05 (0.96, 1.15)	0.299	1758 / 128	1.09 (0.873 , 1.37)	0.436	2465/128	1.07 (0.87, 1.28)	0.502	5663/149	1.02 (0.91, 1.15)	0.707
	**9525/155**	**1.09 (0.99, 1.20)**	0.083	**1707/112**	**1.06 (0.83, 1.36)**	0.622	**2397/115**	**1.13 (0.93, 1.37)**	0.209	**5421/133**	**1.07 (0.94, 1.21)**	0.304
Diagnosis Code[2]												
Low back symptom / complaint (Back syndrome with radiating pain)	9866/174	0.88 (0.76, 1.01)	0.068									
	**9525/155**	**0.94 (0.81, 1.09)**	0.391									
Back syndrome without radiating pain (Back syndrome with radiating pain)	9866/174	1.00 (0.86, 1.16)	0.967									
	**9525/155**	**1.11 (0.95, 1.30)**	0.191									

Results for the univariable analysis were in the corresponding upper row and results for multivariable analysis in the corresponding lower row (grey shaded). If the predictor was not considered in multivariable analysis, the fields are blank. Multivariable analysis for LBP subgroups included predictors of the overall model for all patients. Bold: Significant results are presented in bold.

Supplementary Material, Table S7: Co-medications in 10,331 patients with a low back pain diagnosis.

	Total	Back syndrome with radiating pain	Back syndrome without radiating pain	Low back symptom / complaint	Combination of symptoms	*p*
	N = 10,331	*N* = 1758	*N* = 2465	*N* = 5663	*N* = 445	
Patients without any additional medication	6405 (62.0)	958 (54.5)	1593 (64.6)	3639 (64.3)	215 (48.3)	
Patients with any additional medication	3926 (38.0)	800 (45.5)	872 (35.4)	2024 (35.7)	230 (51.7)	**<0.001**
Patients receiving	*N* = 3926	*N* = 800	*N* = 872	*N* = 2024	*N* = 230	
• PPI	2380 (60.6)	559 (69.9)	503 (57.7)	1178 (58.2)	140 (60.9)	**<0.001**
• Sleeping pills	715 (18.2)	162 (20.2)	154 (17.7)	348 (17.2)	51 (22.2)	0.157
• Antidepressants	826 (21.0)	160 (20.0)	217 (24.9)	409 (20.2)	40 (17.4)	**0.011**
• Muscle relaxants	1312 (33.4)	218 (27.3)	289 (33.1)	715 (35.3)	90 (39.1)	**<0.001**
Patients with additional ICPC-2 Codes receiving[1]	*N* = 207	*N* = 49	*N* = 57	*N* = 89	*N* = 12	
• PPI	68 (32.9)	14 (28.6)	23 (40.4)	26 (29.2)	5 (41.7)	0.303
• Sleeping pills	58 (28.0)	15 (30.6)	15 (26.3)	24 (27.0)	4 (33.3)	0.902
• Antidepressants	95 (45.9)	24 (49.0)	25 (43.9)	40 (44.9)	6 (50.0)	0.867
• Muscle relaxants	3 (1.4)	0 (0.0)	0 (0.0)	3 (3.3)	0 (0.0)	0.135
Patients with NSAID and additional pain medications (relative)	2811 (50.7)	595 (58.7)	638 (43.2)	1410 (50.7)	168 (61.3)	**<0.001**
• Any medication	*N* = 2811	*N* = 595	*N* = 638	*N* = 1410	*N* = 168	
• PPI	1760 (62.6)	437 (73.4)	380 (59.6)	834 (59.1)	109 (64.9)	**<0.001**
• Sleeping pills	459 (16.3)	105 (17.6)	105 (16.5)	224 (15.9)	25 (14.9)	0.623
• Antidepressants	521 (18.5)	106 (17.8)	138 (21.6)	249 (17.7)	28 (16.7)	0.085
• Muscle relaxants	1096 (39.0)	189 (31.8)	238 (37.3)	588 (41.7)	81 (48.2)	**<0.001**
Patients with paracetamol and additional pain medications (relative)	1436 (56.4)	305 (69.2)	275 (53.7)	754 (52.4)	102 (67.1)	**<0.001**
• Any medication	*N* = 1436	*N* = 305	*N* = 275	*N* = 754	*N* = 102	
• PPI	900 (62.7)	211 (69.2)	164 (59.6)	456 (60.5)	69 (67.6)	**0.018**
• Sleeping pills	304 (21.2)	73 (23.9)	58 (21.1)	146 (19.4)	27 (26.5)	0.249
• Antidepressants	345 (24.0)	75 (24.6)	84 (30.5)	165 (21.9)	21 (20.6)	**0.016**
• Muscle relaxants	518 (36.1)	106 (34.8)	84 (30.5)	286 (37.9)	42 (41.2)	0.085
Patients with opioids and additional pain medications	911 (64.3)	250 (68.1)	163 (58.6)	418 (64.1)	80 (66.7)	**0.045**

(relative)						
• Any medication	*N* = 911	*N* = 250	*N* = 163	*N* = 418	*N* = 80	
• PPI	597 (65.5)	183 (73.2)	96 (58.9)	266 (63.6)	52 (65.0)	**0.006**
• Sleeping pills	190 (20.9)	58 (23.2)	36 (22.1)	81 (19.4)	15 (18.8)	0.472
• Antidepressants	211 (23.2)	56 (22.4)	53 (32.5)	87 (20.8)	15 (18.8)	**0.010**
• Muscle relaxants	359 (39.4)	90 (36.0)	70 (42.9)	162 (38.8)	37 (46.2)	0.367
List of all prescribed PPI	*N* = 2252	*N* = 529	*N* = 468	*N* = 1122	*N* = 133	
• Omeprazole	192 (8.5)	49 (9.3)	55 (11.8)	70 (6.2)	18 (13.5)	**0.001**
• Pantoprazole	1854 (82.3)	436 (82.4)	371 (79.3)	943 (84.0)	104 (78.2)	0.079
• Lansoprazole	59 (2.6)	9 (1.7)	15 (3.2)	27 (2.4)	8 (6.0)	0.320
• Rabeprazole	8 (0.4)	1 (0.2)	4 (0.9)	1 (0.1)	2 (1.5)	**0.030**
• Esomeprazole[2]	231 (10.2)	52 (9.8)	50 (10.7)	118 (10.5)	11 (8.3)	0.897
List of all prescribed sleeping pills	*N* = 627	*N* = 141	*N* = 131	*N* = 312	*N* = 43	
• Lorazepam	320 (51.0)	72 (51.1)	64 (48.9)	166 (53.2)	18 (41.9)	0.699
• Alprazolam	43 (6.9)	5 (3.5)	10 (7.6)	25 (8.0)	3 (7.0)	0.203
• Triazolam	13(2.1)	4 (2.8)	0 (0.0)	8 (2.6)	1 (2.3)	0.187
• Temazepam	3 (0.5)	0 (0.0)	3 (2.3)	0 (0.0)	0 (0.0)	**0.010**
• Zolpidem	270 (43.1)	64 (45.4)	60 (45.8)	127 (40.7)	19 (44.2)	0.490
• Dexepin	1 (0.2)	0 (0.0)	0 (0.0)	1 (0.3)	0 (0.0)	1.000
• Diphenhydramine	17 (2.7)	3 (2.1)	4 (3.1)	7 (2.2)	3 (7.0)	0.876
• Doxylamine	20 (3.2)	4 (2.8)	3 (2.3)	12 (3.8)	1 (2.3)	0.698
List of all prescribed antidepressants	*N* = 704	*N* = 129	*N* = 185	*N* = 357	*N* = 33	
• Imipramine	1 (0.1)	0 (0.0)	1 (0.5)	0 (0.0)	0 (0.0)	0.471
• Clomipramine	5 (0.7)	1 (0.8)	2 (1.1)	1 (0.3)	1 (3.0)	0.639
• Trimipramine	162 (23.0)	18 (14.0)	39 (21.1)	97 (27.2)	8 (24.2)	**0.009**
• Amitriptyline	38 (5.4)	15 (11.6)	8 (4.3)	14 (3.9)	1 (3.0)	**0.004**
• Nortriptyline	1 (0.1)	0 (0.0)	0 (0.0)	1 (0.3)	0 (0.0)	1.000
• Maprotiline	10 (1.4)	5 (3.9)	2 (1.1)	3 (0.8)	0 (0.0)	**0.036**
• Fluoxetine	43 (6.1)	6 (4.7)	9 (4.9)	27 (7.6)	1 (3.0)	0.361
• Citalopram	84 (11.9)	11 (8.5)	28 (15.1)	41 (11.5)	4 (12.1)	0.194
• Paroxetine	23 (3.3)	3 (2.3)	6 (3.2)	11 (3.1)	3 (9.1)	0.915
• Sertraline	60 (8.5)	11 (8.5)	18 (9.7)	30 (8.4)	1 (3.0)	0.879
• Fluvoxamine	1 (0.1)	0 (0.0)	0 (0.0)	1 (0.3)	0 (0.0)	1.000
• Trazodone	143 (20.3)	33 (25.6)	37 (20.0)	67 (18.8)	6 (18.2)	0.264
• Mirtazapine	149 (21.2)	29 (22.5)	41 (22.2)	66 (18.5)	13 (39.4)	0.499
• Bupropion	14 (2.0)	2 (1.6)	6 (3.2)	6 (1.7)	0 (0.0)	0.444
• Venlafaxine	82 (11.6)	12 (9.3)	21 (11.4)	44 (12.3)	5 (15.2)	0.658

List of all prescribed muscle relaxants	*N* = 1271	*N* = 207	*N* = 278	*N* = 698	*N* = 88	
• Baclofen	6 (0.5)	1 (0.5)	2 (0.7)	3 (0.4)	0 (0.0)	0.849
• Tizanidine	1237 (97.3)	197 (95.2)	269 (96.8)	686 (98.3)	85 (96.6)	**0.040**
• Diazepam	39 (3.1)	13 (6.3)	9 (3.2)	11 (1.6)	6 (6.8)	**0.003**

[1]: patients with ICPC-2 codes, which would qualify for the treatment with one of our selected co-medications. These patients were excluded for further analysis. Full list of all relevant ICPC-2 codes is provided within the supplemental. Bold: Significant results are presented in bold.

[2]: include esomeprazole alone and fixed combination with Naproxen.

- List of all ICPC-2 Codes, which would qualify for a pain medication: A01 Pain general/multiple sites, A79 Malignancy NOS, A80 Trauma/injury NOS, A81 Multiple trauma/injuries, F01 Eye pain , H01 Ear pain/earache, H70 Otitis externa, K01 Heart pain, K03 Cardiovascular pain NOS, L01 Neck symptom/complain, L02 Back symptom/complaint, L05 Flank/axilla symptom/complaint, L07 Jaw symptom/complaint, L08 Shoulder symptom/complaint, L09 Arm symptom/complaint, L10 Elbow symptom/complaint, L11 Wrist symptom/complaint, L12 Hand/finger symptom/complaint, L13 Hip symptom/complaint, L14 Leg/thigh symptom/complaint, L15 Knee symptom/complaint, L16 Ankle symptom/complaint, L17 Foot/toe symptom/complaint, L18 Muscle pain, L19 Muscle symptom/complaint NOS, L20 Joint symptom/complaint NOS, L26 Fear of cancer musculoskeletal, L27 Fear musculoskeletal disease other, L28 Limited function/disability (l), L29 Sympt/complt. Musculoskeletal other, L70 Infections musculoskeletal system, L71 Malignant neoplasm musculoskeletal, L72 Fracture: radius/ulna, L73 Fracture: tibia/fibula, L74 Fracture: hand/foot bone, L75 Fracture: femur, L76 Fracture: other, L77 Sprain/strain of ankle, L78 Sprain/strain of knee, L79 Sprain/strain of joint NOS, L80 Dislocation/subluxation, L81 Injury musculoskeletal NOS, L82 Congenital anomaly musculoskeletal, L83 Neck syndrome, L85 Acquired deformity of spine, L87 Bursitis/tendinitis/synovitis NOS, L88 Rheumatoid/seropositive arthritis, L89 Osteoarthrosis of hip, L90 Osteoarthrosis of knee, L91 Osteoarthrosis other, L92 Shoulder syndrome, L93 Tennis elbow, L94 Osteochondrosis, L95 Osteoporosis, L96 Acute internal damage knee, L97 Neoplasm benign/unspec musculo., N01 Headache, N03 Pain face, N71 Meningitis/encephalitis, N89 Migraine, N90 Cluster headache, N91 Facial paralysis/bell's palsy, N92 Trigeminal neuralgia, N93 Carpal tunnel syndrome, N95 Tension headache, R01 Pain respiratory system, R72 Strep throat, R73 Boil/abscess nose, R75 Sinusitis acute/chronic, R84 Malignant neoplasm bronchus/lung, R85 Malinant neoplasm respiratory, other, S01 Pain/tenderness of skin, S09 Infected finger/toe, S10 Boil/carbuncle, S11 Skin infection post-traumatic, S70 Herpes zoster, T92 Gout, U01 Dysuria/painful urination, U70 Pyelonephritis/pyelitis, U71 Cystitis/urinary infection other, U75 Malignant neoplasm of kidney, U76 Malignant neoplasm of bladder, U77 Malignant neoplasm urinary other, X01 Genital pain female, X02 Menstrual pain, X03 Intermenstrual pain, X04 Painful intercourse female, X75 Malignant neoplasm cervix, X76 Malignant neoplasm breast female, X77 Malignant neoplasm genital other (f), X78 Fibromyoma uterus, Y01 Pain in penis, Y02 Pain in testis/scrotum , Y77 Malignant neoplasm prostate, Y78 Malign neoplasm male genital other, Y80 Injury male genital, A03 Fever, A75 Infectious mononucleosis, B70 Lymphadenitis acute, B71 Lymphadenitis non-specific, B72 Hodgkin's disease/lymphoma, B73 Leukaemia, B74 Malignant neoplasm blood other, B76 Ruptured spleen traumatic, D01 Abdominal pain/cramps general, D04 Rectal/anal pain, D71 Mumps, D74 Malignant neoplasm stomach, D75 Malignant neoplasm colon/rectum, D76 Malignant neoplasm pancreas, D77 Malig. neoplasm digest other/NOS, D78 Neoplasm digest benign/uncertain, D88 Appendicitis, D92 Diverticular disease, D95 Anal fissure/perianal abscess, F70 Conjunctivitis infectious, F71 Conjunctivitis allergic, F72 Blepharitis/stye/chalazion, F73 Eye infection/inflammation other, K96 Haemorrhoids, N74 Malignant neoplasm nervous system, N75 Benign neoplasm nervous system, N76 Neoplasm nervous system unspec, N80 Head injury other, R21 Throat symptom/complaint, R74 Upper respiratory infection acute, R81 Pneumonia, R82 Pleurisy/pleural effusion, S14 Burn/scald, S16 Bruise/contusion, S77 Malignant neoplasm of skin, S94 Ingrowing nail, U08 Urinary retention, X09 Premenstrual symptom/complaint, X16 Vulval symptom/complaint, X18 Breast pain female, Y73 Prostatitis/seminal vesiculitis, Y74 Orchitis/epididymitis, Y75 Balanitis

- List of all ICPC-2 Codes, which would qualify for a proton pump inhibitor: D03 Heartburn, D07 Dyspepsia/indigestion, D14 Haematemesis/vomiting blood, D15 Melaena, D73 Gastroenteritis presumed infection, D84 Oesophagus disease, D85 Duodenal ulcer, D86 Peptic ulcer other

- List of all ICPC-2 Codes, which would qualify for sleeping pills: P01 Feeling anxious/nervous/tense, P02 Acute stress reaction, P74 Anxiety disorder/anxiety state, P79 Phobia/compulsive disorder, P06 Sleep disturbance, N88 Epilepsy, N04 Restless legs, P15 Chronic alcohol abuse, P16 Acute alcohol abuse

- List of all ICPC-2 Codes, which would qualify for antidepressants: P76 Depressive disorder, P03 Feeling depressed, P01 Feeling anxious/nervous/tense, P74 Anxiety disorder/anxiety state, L18 Muscle pain, L19 Muscle symptom/complaint NOS, N89 Migraine, X11 Menopausal symptom/complaint, P79 Phobia/compulsive disorder, P17 Tobacco abuse, P81 Hyperkinetic disorder, P82 Post-traumatic stress disorder, P07 Sexual desire reduced, P08 Sexual fulfilment reduced, Y07 Impotence NOS, Y08 Sexual function sympt./complt.(m), P86 Anorexia nervosa/bulimia

- List of all ICPC-2 Codes, which would qualify for antispasmodic medications: N86 Multiple sclerosis

List of all used ATC codes for the identification of pain medications:

**Pain medications**		
**NSAIDS (M01A)**		
	Celecoxib	M01AH01
	Diclofenac	M01AB05
	Diclofenac topical	M02AA15
	Diclofenac combinations	M01AB55
	Etodolac	M01AB08
	Ibuprofen	M01AE01
	Ibuprofen topical	M02AA13
	Ibuprofen combinations	M01AE51
	Ibuprofen + Oxicodone	N02AJ19
	Indomethacin	M01AB01
	Mefenamic Acid	M01AG01
	Naproxen	M01AE02
	Naproxen topical	M02AA12
	Naproxen + Esomeprazole	M01AE52
	Naproxen + Misoprostol	M01AE56
	Ketorolac	M01AB15
**Paracetamol**		
	Paracetamol	N02BE01
	Paracetamol combinations	N02BE71
**Opioids (N02A)**		
Weak Opioids	Dihydrocodeine	N02AA08
	Codeine (combinations)	N02AA59
	Tilidine	N02AX01
	Tramadol	N02AX02
	Tapentadol	N02AX06
	Tramadol combinations	N02AJ15
	Tramadol paracetamol	N02AJ13
Strong Opioids	Morphine	N02AA01
	Morphine combinations	N02AA51
	Hydromorphone	N02AA03
	Hydromorphone combinations	N02AA53
	Nicomorphine	N02AA04
	Oxycodone	N02AA05
	Oxycodone + ASS	N02AJ18
	Oxycodone + Ibuprofen	N02AJ19
	Oxycodone +Paracetamol	N02AJ17
	Oxycodone + Naloxon	N02AA55
	Pethidine	N02AB02
	Pethidine combinations	N02AG03
	Fentanyl systemic	N01AH01
	Fentanyl topic	N02AB03

	Fentanyl combinations	N01AH51
	Buprenorphine	N02AE01
	Nalbuphine	N02AF02
	Methadone combinations	N02AC52
	Piritramid	N02AC03

List of all used ATC codes for the identification of co-medications:

**Co - medications**		
**Muscle relaxants (M03)**		
Antispasticity medications	Baclofen	M03BX01
	Dantrolene	M03CA01
Antispasmodic medications	Metaxalone	
	Carisoprodol	M03BA02<br>M03BA52 (combinations)<br>M03BA72 (combinations)
	Chlorzoxazone	M03BB03 (single)<br>M03BB53 (combinations)<br>M03BB73 (combinations)
	Cyclobenzaprine	M03BX08
	Methocarbamol	M03BA03<br>M03BA53 (combinations)<br>M03BA73 (combinations)
	Orphenadrine	N04AB02 (chloride)<br>M03BC01 (citrate)<br>M03BC51 (combinations)
Antispasticity and Antispasmodic Agents	Tizanidine	M03BX02
	Diazepam	N05BA01
**Proton pump inhibitors(A02BC)**		
	Omeprazole	A02BC01
	Pantoprazole	A02BC02
	Lansoprazole	A02BC03
	Rabeprazole	A02BC04
	Esomeprazole	A02BC05
	Vonoprazan	A02BC08
**Laxatives**		
Drugs for constipation		A06A
Propulsives		A03F
**Sleeping pills**		
Sedating antihistamines	Diphenhydramine	R06AA02
	Doxylamine	R06AA09
	Cyclizine	R06AE03
Hypnotics	Zolpidem	N05CF02
	Zaleplon	N05CF03
	Eszopiclone	N05CF04
	Ramelteon	N05CH02
Benzodiazepines	Alprazolam	N05BA12
	Diazepam	N05BA01
	Lorazepam	N05BA06
	Triazolam	N05CD05
	Estazolam	N05CD04
	Temazepam	N05CD07

Tricyclic antidepressiva	Doxepin	N06AA12
other	Suvorexant	N05CM19
**Antidepressants (N06A)**		
	Amitriptyline	N06AA09
	Clomipramine	N06AA04
	Doxepin	N06AA12
	Imipramine	N06AA02
	Trimipramine	N06AA06
	Amoxapine	N06AA17
	Desipramine	N06AA01
	Nortriptyline	N06AA10
	Protriptyline	N06AA11
	Maprotiline	N06AA21
	Mirtazapine	N06AX11
	Trazodone	N06AX05
	Bupropion	N06AX12
	Venlafaxine	N06AX16
	Nefazodone	N06AX06
	Fluoxetine	N06AB03
	Paroxetine	N06AB05
	Sertraline	N06AB06
	Citalopram	N06AB04
	Fluvoxamine	N06AB08
	Isocarboxazid	N06AF01
	Phenelzine	N06AF03
	Tranylcypromine	N06AF04
